# The trajectory of intrinsic capacity and its related factors among elderly Chinese patients with cardiovascular disease: a prospective cohort study

**DOI:** 10.3389/fendo.2025.1539982

**Published:** 2025-05-27

**Authors:** Yanyin Cui, Shanyan Lei, Yubing Xia, Fang Yang

**Affiliations:** ^1^ School of Humanities and Management, Zhejiang Chinese Medical University, Hangzhou, China; ^2^ Department of Rehabilitation Medicine, The First Affiliated Hospital, Zhejiang University School of Medicine, Hangzhou, China

**Keywords:** elderly, cardiovascular disease, intrinsic capacity, trajectory, cohort study

## Abstract

**Objectives:**

To track the dynamic trajectory of intrinsic capacity (IC) in elderly patients with cardiovascular disease (CVD), and systematically assess the statistical associations between baseline characteristics, time-varying factors and distinct trajectory groups.

**Methods:**

The data were procured from the China Health and Retirement Longitudinal Study in 2011, 2013, and 2015, encompassing 701 elderly patients with CVD. The Group-based Trajectory Model (GBTM) was used to fit the developmental trajectory of IC in these patients and to identify the types of longitudinal change trajectories of IC. Unordered multinomial logistic regression was utilized to evaluate the strength of the association between non-time-varying baseline characteristics and trajectory classifications, while a panel regression model was applied to analyze the association patterns between time-varying indicators and the dynamic evolution of these trajectories.

**Results:**

The GBTM model identified three categories of IC trajectories among elderly patients with CVD: the low-decreased group (n=162, 23.11%), the high-increased group (n=196, 27.96%), and the medium-stable group (n=343, 48.93%). The classification of the IC trajectories in elderly CVD patients is connected with baseline characteristics and time-varying factors. The analysis of associated factors show that non-time-varying characteristics such as educational level, residential area, housing type, toilet facilities, and energy sources, are significantly associated with the IC trajectories of CVD patients. Marital status, masticatory function, sleep disorders, chronic pain, comorbid chronic diseases, and the diversity of social activities exhibit a dynamic association with the evolution of the IC trajectory patterns.

**Conclusions:**

IC development in elderly patients with CVD is characterized by both group heterogeneity and individual variability. Consequently, healthcare providers can develop personalized health management programs based on the characteristics of different patients.

## 1 Introduction

Cardiovascular disease (CVD) is a common disease that significantly threatens human health, especially among middle-aged and elderly people over 50 years of age. It is the leading cause of mortality worldwide (1). The annual report on cardiovascular health and diseases in China (2023) demonstrated a persistent and ongoing increase in the prevalence of CVD in China (2). CVD represents the primary cause of disease-related mortality among urban and rural residents in China, with the aging process being the predominant contributing factor. China currently has the largest number of elderly people over 65 years of age globally. Moreover, the degree and pace of aging continue to increase, with China truly entering a deeply aging society. The prevalence and mortality rates of CVD in China are anticipated to rise further. CVD constitutes a significant and prevalent health concern among the elderly, with the potential to significantly impact the health and well-being of this demographic. Aging leads to long-term survival with disease and an increasing cardiometabolic burden, which invariably exacerbates the challenges of CVD prevention and control (3). In this context, identifying and mastering the health change characteristics in elderly patients with CVD is crucial for strengthening the prevention and control mechanisms of CVD, as well as for improving the quality of life and survival outcomes within this vulnerable population.

In the context of an aging population, shifting the focus from the traditional disease diagnosis and treatment toward maintaining the functional status of older adults, especially their intrinsic capacity (IC), has become the key to achieving active and healthy aging. In 2015, the World Health Organization (WHO) introduced the concept of IC, which is defined as the combination of all the mental and physical abilities that an individual can use and contains six key domains: Vision, hearing, psychology, vitality, locomotion, and cognition (4, 5). The WHO advocates for the monitoring of the IC trajectory of older adults as a strategy to prevent adverse health outcomes and delay health deterioration. Recent suggestions propose that assessing the health status of elderly patients with CVD through the lens of IC may redirect the focus of health management from disease treatment to the enhancement of IC (6, 7). This approach may effectively capture the heterogeneity of health within this population and contribute to the preservation of IC in older patients with CVD, thereby preventing adverse health outcomes. IC is a fundamental attribute that is connected with an older person’s ability to age healthily and maintain functional independence. It encompasses the full range of an individual’s physiological functions and cognitive abilities available for use at any given time. IC is shaped by the interplay of personal attributes, health-related factors, and other determinants (4). Pertinent research indicates that genetic factors account for 25% of IC in older adults, while 75% is related to lifestyle behaviors and the extent of interaction with and intervention in the external environment (8). Furthermore, studies demonstrate a significant association between IC in older adults and various factors, including demographic, socioeconomic, disease-related, and behavioral factors (9).

IC is a dynamic and variable process that reflects alterations in functional trajectories throughout the lifespan. The developmental trajectories of intrinsic capacity (IC) among elderly patients with CVD may evolve over time, with the characteristics of dynamic trajectories and the exploration of trajectory heterogeneity increasingly becoming a focal point of research. Current studies on IC trajectories have an established academic foundation in general elderly populations. For instance, a study conducted in Mexico classified IC trajectories in older adults into categories of rapid decline, moderate decline, and slight increase (10, 11); a South Korean community-based study identified four IC trajectory patterns: low-persistent, low-increasing, high-decreasing, and high-stable (11); while a Chinese community cohort study classified elderly IC trajectories as rapid decline, moderate decline, and mild decline (12). Multinational cohort studies suggest that elderly IC can generally be categorized into high, medium, and low levels, with identifiable “stable”, “fluctuating”, and “progressive” patterns, demonstrating commonality while exhibiting significant population heterogeneity in IC trajectories. Notably, research indicates that cohort trends of IC in older adults show broad consistency between the UK and China, further supporting the existence of common patterns in IC decline trajectories among aging populations (13).

IC trajectories are often strongly correlated with negative health outcomes (e.g., disability, mortality, hospitalization rates) (14–16). Consequently, identifying the characteristics of IC and its trajectories of change in elderly patients with CVD is crucial for proactively addressing health issues in this population. This understanding is also essential for developing personalized, multidomain intervention models tailored to elderly patients with CVD. While existing research primarily investigate IC evolution patterns in community-dwelling older adults, there is a significant gap in research focused on CVD populations. Elderly CVD patients experience a more rapid decline in IC compared to the general aging population, which is connected with the combined effects of pathophysiological burden and treatment-related side effects. Elderly patients with CVD exhibit lower IC compared to the general elderly population. Investigating and enhancing these factors can effectively improve their overall function and offer a novel approach to developing a health management model for elderly patients with CVD (17). Nevertheless, there is a lack of research specifically targeting elderly patients with CVD, with most existing studies concentrating on isolated dimensions of IC, such as cognitive (18), psychological (19), or nutritional aspects (20). Consequently, comprehensive analyses examining the developmental trajectory of IC in its entirety, along with its influencing factors, are scarce.

To address these research gaps, this study utilized a nationally representative database to investigate IC status among elderly Chinese patients with CVD. This study utilizes Group-based Trajectory Modeling (GBTM) to identify potential categories of IC developmental trajectories in elderly patients with CVD and to analyze the factors that correlate with these latent classes. The primary objectives are to identify subgroups experiencing rapid decline for targeted interventions, enabling healthcare professionals to conduct comprehensive assessments of patients’ disease status, and providing evidence-based guidance for determining optimal timing of integrated care services and implementing precise intervention models tailored to elderly patients with CVD.

## 2 Materials and methods

### 2.1 Sample and data source

#### 2.1.1 Sample selection

The data and samples used in this study were procured from the China Health and Retirement Longitudinal Survey (CHARLS), a large-scale, long-term tracking survey project conducted by the China Centre for Social Science Research at Peking University. The database employs a multi-stage probability proportional to size sampling method to conduct household surveys targeting middle-aged and elderly populations across 28 provincial-level administrative regions in China. This encompasses 150 county-level units and 450 village-level units. The questionnaires are designed in accordance with international standards, achieving an interview response rate and data quality that rank among the highest globally for comparable projects. Consequently, the data have been extensively utilized and acknowledged within the academic community.

**Figure 1:**
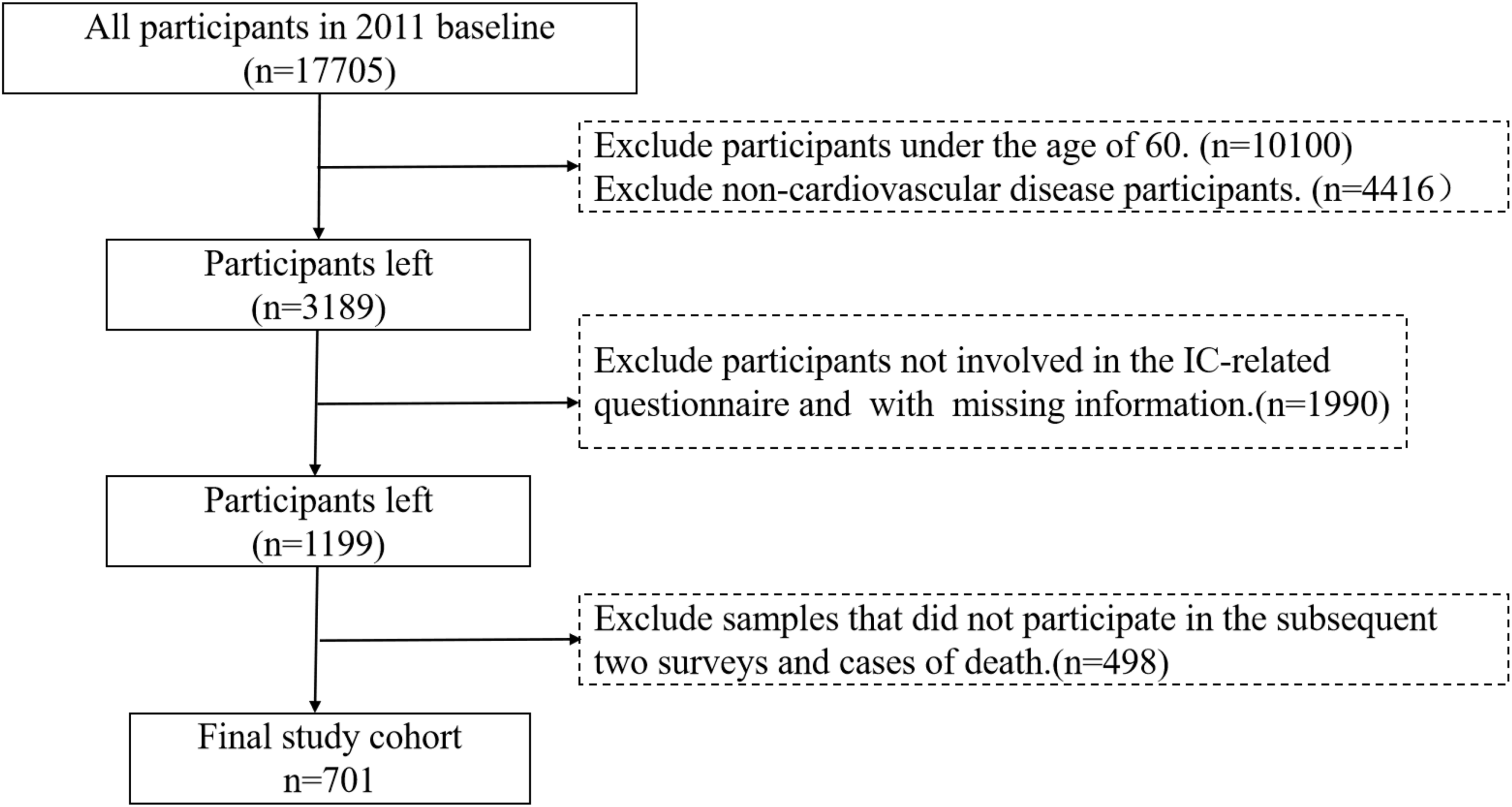
Data extraction and management flowchart.

This study utilizes data from CHARLS 2011, 2013, and 2015 cohorts, which include patients with CVD aged 60 and older. To meet the requirements for IC trajectory analysis, a total of 701 valid cases were obtained following data processing (see **Figure 1** for screening workflow). Based on CVD definitions and CHARLS questionnaires, core diagnostic inclusion criteria were established, with inclusion qualifying upon meeting any of the following criteria: (1) Hypertension: systolic blood pressure ≥140 mmHg or diastolic blood pressure ≥90 mmHg, or current use of antihypertensive medication; (2) Self-reported or physician-diagnosed myocardial infarction, coronary heart disease, angina pectoris, congestive heart failure, or other cardiac conditions. Dyslipidemia was considered as a comorbid risk factor rather than an independent inclusion criterion.

#### 2.1.2 Sample representativeness analysis

The primary reason for the reduction in sample size during data completeness screening (Step 3) was the high attrition rate observed in elderly cohorts (e.g., death, loss to follow-up, refusal to respond), consistent with similar longitudinal studies (e.g., the Survey of Health, Ageing and Retirement in Europe [SHARE] and the Health and Retirement Study [HRS]) (21, 22). The selection of participants with three consecutive waves of complete dataadheres to the methodological requirements of Group-based Trajectory Modeling (GBTM), which necessitates continuous observations across at least three time points. To address potential concerns about the representativeness ofthe 701-sample cohort, the following validity verification was performed.

A sensitivity analysis comparing baseline characteristics between three-wave participants (*n*=701) and 1–2 wave participants (*n*=535) revealed no significant differences in multimorbidity, major accidental injuries, or chronic pain (*p* > 0.05) (see **Table 1**). However, attrited participants exhibited a higher baseline age and a greater prevalence of severe functional impairment (1.788 vs. 1.403, *p* = 0.001). This suggests that the attrited group may include individuals with more rapid functional decline, which could correlate with anunderestimation of IC decline rates. Nevertheless, the primary findings remain applicable to the “traceable elderly CVD population”.

**Table 1:** Baseline characteristics of participants with 1–2 waves versus 3 waves.

Characteristic	3 wave participants (*n*=701)	1–2 wave participants (*n*=535)	*P-value*
Age (x ± s)	66.91 ± 5.134	68.235 ± 6.417	0.000
Male	402	267	0.011
Chronic disease co-morbidity level	2.577 ± 1.444	2.674 ± 1.395	0.835
Major accidental injury	65	42	0.415
Chronic pain	221	192	0.114
Number of decreased IC	1.403 ± 1.027	1.788 ± 1.202	0.001

Additionally, a comparison of baseline characteristics was conducted between the final sample (*n*=701) and all CHARLS baseline database entries for cardiovascular patients aged 60 and older with complete data (*n*=2975) (see **Table 2**). The results demonstrated high consistency in demographic and health profiles between the final sample and the original population (*p* > 0.05), with the exception of educational attainment. Therefore, the final sample effectively is considered to be a representative subset of elderly CVD patients within the CHARLS database.

**Table 2:** Key characteristics of the total elderly CVD patient cohort versus the screened population.

Characteristic	Screened cohort (*n*=701)	Elderly CVD patients (*n*=2975)	*P-value*
Age (x ± s)	66.191 ± 5.134	66.839 ± 5.800	0.007
Male	402	1769	0.286
Educational status (≥Junior high school)	486	1689	0.000
Married	596	2492	0.457
Major accidental injury	65	286	0.830
Toothless	75	394	0.078
Chronic pain	221	856	0.153
Sleep duration (hours)	6.287 ± 1.641	6.254 ± 1.853	0.666

### 2.2 Variable measurement

#### 2.2.1 Dependent variable and quantification

In this study, the dependent variable was the composite score representing the level of intrinsic competence in elderly patients with cardiovascular conditions. IC was evaluated based on the six key domains outlined by the WHO in 2019, using the recommended assessment instruments (23). The Simple Physical Performance Battery (SPPB) was employed to evaluate locomotion function, while Body Mass Index (BMI) served as a measure of vitality. The abbreviated version of the Center for Epidemiologic Studies Depression Scale-10 (CESD-10) was utilized to assess psychological function. Cognitive function was assessed using the Mini-Mental State Examination (MMSE). Additionally, sensory function, encompassing both visual and auditory aspects, was evaluated through subjective questioning. Any detected abnormality within these dimensions was interpreted as a diminution in IC.

Research has demonstrated that the functions of the sub-domains of IC are intricately interconnected and can be integrated through potential interaction mechanisms (24). The Criteria Importance Though Intercrieria Correlation (CRITIC) assignment method quantifies the informational value of indicators by assessing the comparative strength and conflict among evaluation indicators. This approach effectively minimizes informational redundancy among indicators, thereby yielding more reliable results (25). The Technique for Order Preference by Similarity to an Ideal Solution (TOPSIS) method incorporates a distance-based measure to define the target space. It evaluates the closeness to the optimal value and the proximity to the worst value, which are connected with the magnitude of the sample score (26). Consequently, this study employed the CRITIC-TOPSIS modeling approach to compute the composite score of IC. The following outlines the formulas and procedures used to construct IC composite scores:

Domain-specific IC indicators were normalized in the positive direction using min-max scaling to constrain values within the range [0,1]. Let $x_{ij}$ denote the raw value of indicator *j* for participant *i*. The normalized value $x_{ij}$ was computed as follows (Equation 1):


(1) $$\begin{array}{c} x{'}_{ij}=\frac{max\left\{x_{j}\right\}-x_{ij}}{max\left\{x_{j}\right\}-min\left\{x_{j}\right\}}\;\left(negative\;indicator\right);\\ x{'}_{ij}=\frac{x_{ij}-min\left\{x_{j}\right\}}{max\left\{x_{j}\right\}-min\left\{x_{j}\right\}}\;\left(positive\;indicator\right) \end{array}$$

Objective weights for IC indicators were determined using the CRITIC method (**Table 3**). Let $\mu_{j}$ represent the mean of indicator *j*. The standard deviation (contrast intensity) $\sigma_{j}$ (Equation 2), correlation matrix construction $\gamma_{jk}$ (Equation 3), conflict index calculation $C_{j}$ (Equation 4), and information content quantification $I_{j}$ (Equation 5) were calculated respectively. The specific weight assignment $\omega_{j}$ (Equation 6) was calculated finally. The computational steps include:

**Table 3:** CRITIC results for each dimension of IC.

Variables	Indicator variability	Indicator conflict	Quantity of information	Weights
Sensory	0.2873	3.5379	1.0165	0.3094
Psychology	0.1973	3.4401	0.6786	0.2066
Vitality	0.0552	4.0179	0.2219	0.0676
Locomotion	0.2108	3.6518	0.7698	0.2343
Cognition	0.1676	3.5710	0.5984	0.1822


(2) $$\sigma_{j}=\sqrt{\frac{1}{n}\sum_{i=1}^{n}{\left(Y_{ij}-\mu_{j}\right)}^{2}}$$

(3) $$\gamma_{jk}=\frac{\sum_{i=1}^{n}\left(Y_{ij}-\mu_{j}\right)\left(Y_{ik}-\mu_{k}\right)}{\sqrt{\sum_{i=1}^{n}{\left(Y_{ij}-\mu_{j}\right)}^{2}\sqrt{\sum_{i=1}^{n}{\left(Y_{ik}-\mu_{k}\right)}^{2}}}}$$

(4) $$C_{j}=\sum_{k=1,\;k\neq j}^{6}\left(1-\gamma_{jk}\right)$$

(5) $$I_{j}=\sigma_{j}\times C_{j}$$

(6) $$\omega_{j}=\frac{I_{j}}{\sum_{k=1}^{6}I_{k}}$$

Finally, the comprehensive score of the IC for each sample is calculated based on the TOPSIS model. A weighted matrix is constructed according to the weight values obtained through the CRITIC weighting method $Z_{ij}=Y_{ij}\times \omega_{j}$ , and then the positive/negative ideal solutions are determined. The positive ideal solutions $A^{+}$ is the maximum value of each indicator, that is $A^{+}=max\left(Z_{ij}\right)$ , and the negative ideal solutions $A^{-}$ is the minimum value of each indicator, that is $A^{-}=min\left(Z_{ij}\right)$ . The calculation of the Euclidean distance for the indicators is shown in Equations 7 and 8. Based on this, the comprehensive score is calculated (Equation 9). The score range is [0, 1], with higher scores indicating better internal capability levels. The Euclidean distances to ideal solutions and composite IC score $E_{i}$ are as follows:


(7) $$D_{i}^{+}=\sqrt{\sum_{j=1}^{6}{\left(Z_{ij}-A_{j}^{+}\right)}^{2}}$$

(8) $$D_{i}^{-}=\sqrt{\sum_{j=1}^{6}{\left(Z_{ij}-A_{j}^{-}\right)}^{2}}$$

(9) $$E_{i}=\frac{D_{i}^{-}}{D_{i}^{+}+D_{i}^{-}}$$

The IC composite index, as a multidimensional metric, lacks direct clinical interpretability when relying solely on standardized scores within the 0–1 range. To address this limitation, this study employed a distribution-based approach (27) to quantify the clinical significance of trajectory group differences, thereby anchoring the results in clinical relevance. The proportion of total variance explained by trajectory group differences (Eta-squared) $\eta^{2}$ was calculated using Equation 10, which involves the between-group sum of squares $SS_{between}$ and total sum of squares $SS_{total\;}$ . Effect sizes for multi-group comparisons were evaluated using Equation 11.

The Equation 11 is applicable to the effect size index Cohen’s *f* for multiple comparisons, and the effect size index Cohen’s *d* for pairwise comparisons at the end, as detailed in Equation 12.


(10) $$\eta^{2}=\frac{SS_{between}}{SS_{total}}$$

(11) $$Cohen's\;f=\sqrt{\frac{\eta^{2}}{1-\eta^{2}}}$$

(12) $$Cohen's\;d=\frac{\left|{\text{μ}}_{\text{A}}-{\text{μ}}_{\text{B}}\right|}{{\text{σ}}_{\text{pooled}}}$$

Where $\mu_{A}$ , $\mu_{B}$ represent the group means and $\sigma_{pooled}$ denotes the pooled standard deviation.

#### 2.2.2 Associated factor variables

In addition to socio-demographic characteristics, factors such as organic health status, healthy lifestyle, social security, and residential environment are related to the trajectory of IC development in older patients with CVD.

Socio-demographic characteristics include age (years old), gender (male = 0, female = 1), residence region (villagers = 0, cities and towns = 1), marital status (married = 1, not married = 0), and educational status (no formal education or primary = 1, no formal education or primary = 2, senior high school = 3, college or university = 4).

The health status of the individual includes chronic disease co-morbidity level (1 = 1, 2 = 2, 3 = 3, 4 or more = 4). This study defined chronic diseases by integrating the WHO Chronic Conditions Inventory and the CHARLS questionnaire. The conditions included both CVD (per inclusion criteria) and non-CVD comorbidities, such asmalignancies (e.g., cancers), chronic pulmonary diseases, hepatic disorders, stroke, renal diseases, gastrointestinal diseases, affective/psychiatric disorders, memory-related disorders, arthritis/rheumatism, and asthma. Comorbidity levels were stratified based on the number of affected organ systems and the severity of the diseases. Major accidental injury (yes = 1, no = 0), chronic pain (yes = 1, no = 0), toothless (yes = 1, no = 0), and sleep disorders (yes = 1, no = 0).

Healthy lifestyle factors include napping habits (yes = 1, no = 0), dietary habits (3 meals per day = 1, others = 0), smoking history (yes = 1, no = 0), drinking (drinking = 0, drink more than once a month = 1, drink but less than once a month = 2), and richness of social activities (0 = 0, 1 = 1, 2 = 2, 3 or more = 3).

Social security encompasses health insurance (yes = 1, no = 0) and pension insurance (yes = 1, no = 0).

The residential environment includes housing type (one-story building = 0, multi-story building = 1), toilet type (toilet without a seat = 0, toilet with a seat = 1), primary source of cooking fuels (clean fuels = 0, solid fuels = 1), heating insurance (yes = 1, no = 0), interior temperature (hot = 1, bearable = 2, cold = 3), and handicapped facility (yes = 1, no = 0).

### 2.3 Model method

Group-Based Trajectory Modeling (GBTM) utilizes finite mixture models with maximum likelihood estimation to semi-parametrically analyze population trajectories, assuming that overall developmental patterns follow an unconditional continuous distribution. This approach effectively clusters heterogeneous trajectories while capturing shared developmental characteristics. In comparison with other methods, (e.g., Latent Growth Mixture Model), GBTM demonstrates superior performance in joint estimation of multidimensional indicators (28). Given that IC—the central metric of this study—is a multidimensional composite metric, we applied GBTM to identify heterogeneous IC trajectory patterns among elderly CVD patients. The model specifications are outlined below:

Suppose there are J several different IC development trajectories within the group of elderly patients with CVD, with each trajectory expressed as a polynomial of the time variable (Equation 13):


(13) $$\ln\left(\lambda_{t}^{j}\right)=\beta_{0}^{j}+\beta_{1}^{j}Age_{it}^{1}+\beta_{2}^{j}Age_{it}^{2}+\cdots +\beta_{k}^{j}Age_{it}^{k}+\epsilon_{it}\;j=1,2,3,\cdots ,J$$

Here, $\lambda_{t}^{j}$ is the average incidence rate of individual events observed at time *t* for a specific trajectory category *j*; ${\text{Age}}_{it}$ represents the time variable, which is the age of individual *i* at time *t*; k is the highest degree of the age polynomial, and $\beta_{k}^{j}$ is the corresponding parameter to be estimated. Under the condition independence assumption of the model, the probability of event occurrence for the observed sample *i* in a given trajectory type *j* can be expressed as Equation 14:


(14) $$P^{j}\left(Y_{i}\right)=P\left(Y_{i}|Age_{it},\;j;\beta^{j}\right)=\prod_{t=1}^{T}p\left(Y_{i}|Age_{it},j;\beta^{j}\right)$$

Where P denotes the probability of occurrence and T represents the total number of observations. The most critical aspect of GBTM lies in determining both the optimal number of trajectory groups and their functional forms (e.g., linear, quadratic), which directly affect the model’s accuracy, parsimony, and stability, which are crucial factors influencing subsequent analyses (29).

### 2.4 Statistical analysis

The statistical package for the social sciences software (25.0) and R software were employed for data analysis. Descriptive statistics are presented as mean ± standard deviation, while categorical data are reported as frequencies and percentages. The CRITIC-TOPSIS modeling method was utilized to calculate the IC composite score. Additionally, GBTM was applied to assess the trajectory of changes in IC among elderly patients with CVD. The GBTM process is initiated with a minimal number of subgroups. For each subgroup, the fitting begins with higher-order terms, subsequently incorporating lower-order terms only if the significance of the higher-order terms is found to be insufficient. The following metrics are commonly employed to assess model efficacy (28, 30, 31): (a) Akaike Information Criterion (AIC), Bayesian Information Criterion (BIC), Sample Size-Adjusted BIC (ssBIC), Hannan-Quinn Information Criterion (HQIC), and Consistent AIC (CAIC). A lower absolute value of these metrics indicates a better model fit. (b) The Odds of Correct Classification (OCC) quantifies the ratio of the probability of correct classification for each group. It is generally accepted that an OCC greater than 5 indicates a high classification accuracy. (c) Proportions per Class (PPC) refers to the sample size of each subgroup, which is typically not less than 5% of the total sample. (d) The Assigned Average Posterior Probability reflects the degree of conformity between subgroup members and their assigned trajectory, with a value greater than 0.7 generally regarded as the acceptable standard for model adequacy. A chi-square test or a one-way analysis of variance was employed to compare the patient characteristics across various categories of IC trajectories. Multinomial logistic regression was applied to evaluate the association strength between time-invariant baseline characteristics and trajectory classification. Panel regression models were utilized to investigate the dynamic relationships between time-varying covariates and trajectory morphology, with statistical significance determined at a threshold of *P* < 0.05.

## 3 Results

### 3.1 Baseline information on elderly patients with CVD

In this study, 701 elderly patients with CVD were analyzed. The participant’s mean age was 66.19 ± 5.13 years, ranging from 60 to 90 years. Of these patients, 299 (43.65%) were females and 402 (57.35%) were males. Hypertension was present in 516 individuals (73.6%), while dyslipidemia (elevation of low-density lipoprotein, triglycerides, and total cholesterol, or a decline of high-density lipoprotein) was observed in 211 individuals (30.2%). Moreover, heart disease, encompassing conditions such as myocardial infarction, coronary artery disease, angina, congestive heart failure, and other cardiac disorders, was identified in 262 cases (37.4%). Notably, no significant difference was observed in the types of CVD across the trajectory subgroups (*P* < 0.05). The mean IC scores of the sample were 0.417 ± 0.155 in 2011, 0.422 ± 0.153 in 2013, and 0.405 ± 0.159 in 2015. Further details are presented in **Supplementary Table S1** of the Annex.

### 3.2 Model fit and selection of IC trajectories for elderly patients in CVD

#### 3.2.1 Model fit and selection of IC trajectories for elderly patients in CVD

When the study cohort exhibits significant internal heterogeneity, individual developmental trajectories fail to demonstrate a continuous normal distribution across the group. In such instances, approximate developmental trajectories can be categorized by analyzing individual developmental trajectories using GBTM. In this study, a total of 701 participants were included in the analysis, utilizing the patients’ three-phase IC scores as the observational index. The GBTM was employed to model the developmental trajectory categories of IC, ranging from low to high and from higher to lower order. As demonstrated in **Table 4**, the minimal absolute values of AIC, BIC, ssBIC, HQIC, and CAIC converged for both the 3- and 5-class trajectory models. The 3-class solution exhibited a relatively balanced subgroup distribution (23.11%, 27.96%, 48.93%), which is consistent with the latent functional stratification patterns observed in aging populations. In contrast, the 5-class model included a small minority subgroup (6.0%), indicating a potential for overfitting and limited clinical utility. Consequently, the 3-class IC trajectory model was selected for subsequent analyses.

**Table 4:** Results of GBTM model fitting for trajectories of IC in elderly patients with CVD.

Trajectory model	AIC	BIC	CAIC	ssBIC	HQIC	Minimus OCC	AvePP					PPC%				
							G1	G2	G3	G4	G5	G1	G2	G3	G4	G5
1-Trajectory model	-1837.521	-1826.219	-1824.219	-1832.573	-1833.381	—	1.000	—	—	—	—	100%	—	—	—	—
2-Trajectory model	-2414.765	-2369.56	-2361.56	-2394.977	-2398.209	11.650	0.907	0.932	—	—	—	46.79%	53.21%	—	—	—
3-Trajectory model	-2594.876	-2521.418	-2508.418	-2562.720	-2567.971	18.000	0.888	0.884	0.877	—	—	23.11%	27.96%	48.93%	—	—
4-Trajectory model	-2597.900	-2496.188	-2478.188	-2553.376	-2560.647	14.001	0.879	0.878	0.675	0.693	—	24.11%	31.38%	15.41%	29.10%	—
5-Trajectory mode	-2646.668	-2528.005	-2507.005	-2594.724	-2603.207	21.748	0/891	0.838	0.699	0.692	0.752	23.11%	6.00%	22.11%	22.82%	25.96%

**Figure 2:**
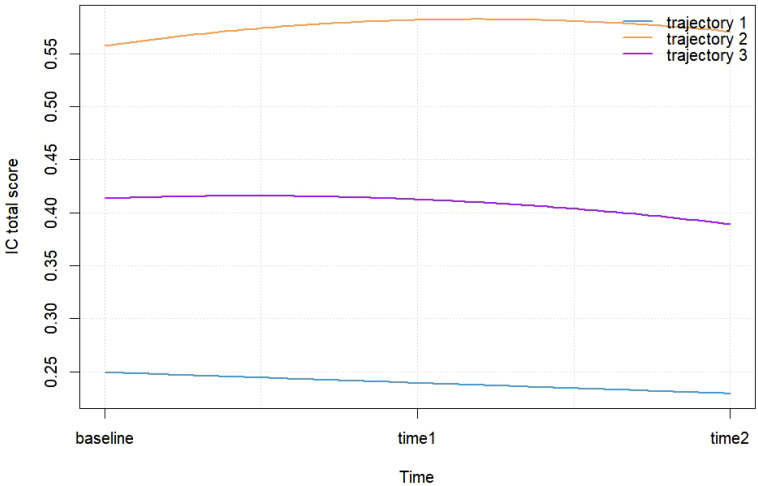
Trajectory categories of IC in elderly patients with CVD.

**Figure 3:**
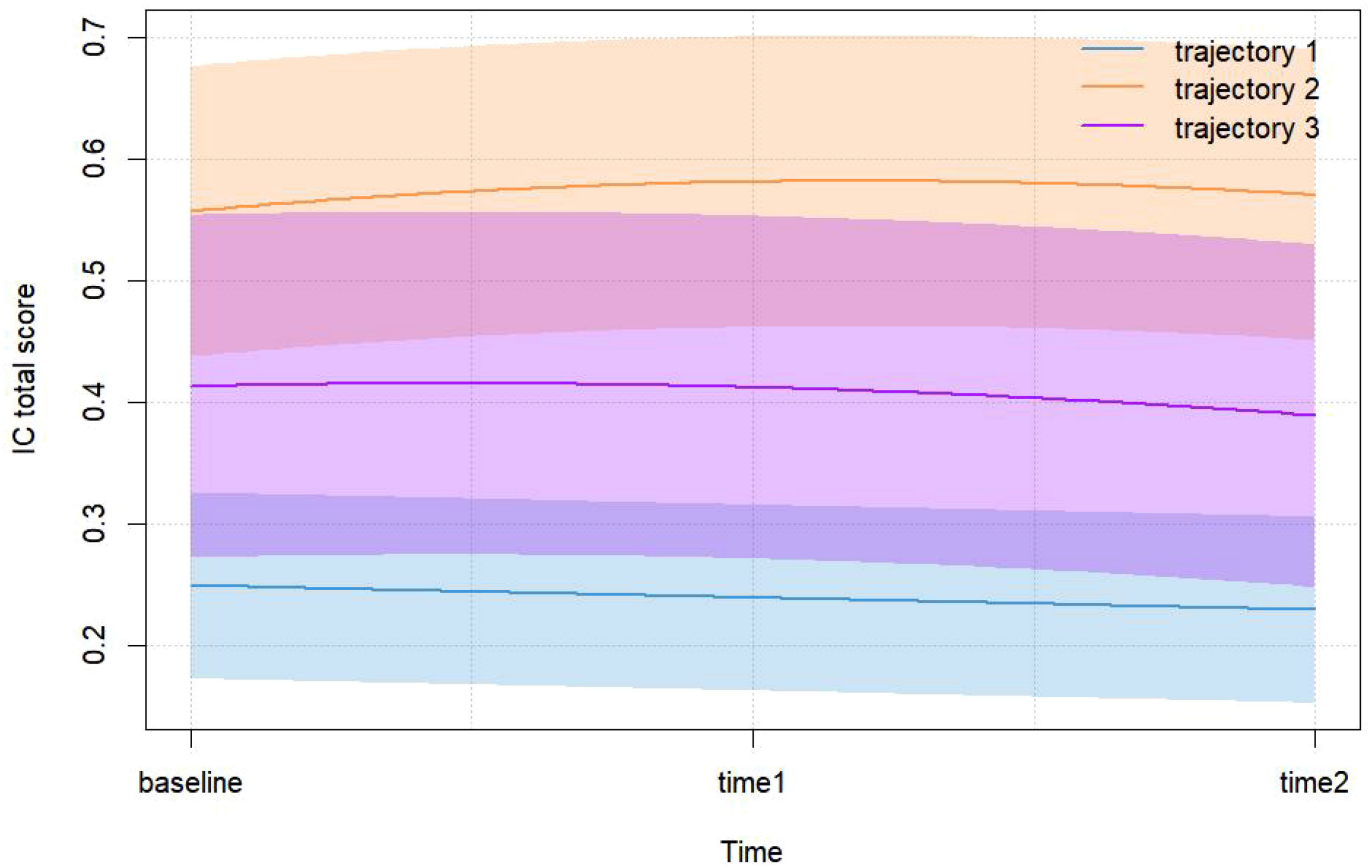
Trajectory categories of IC in elderly patients with CVD (with confidence interva).

**Figure 4:**
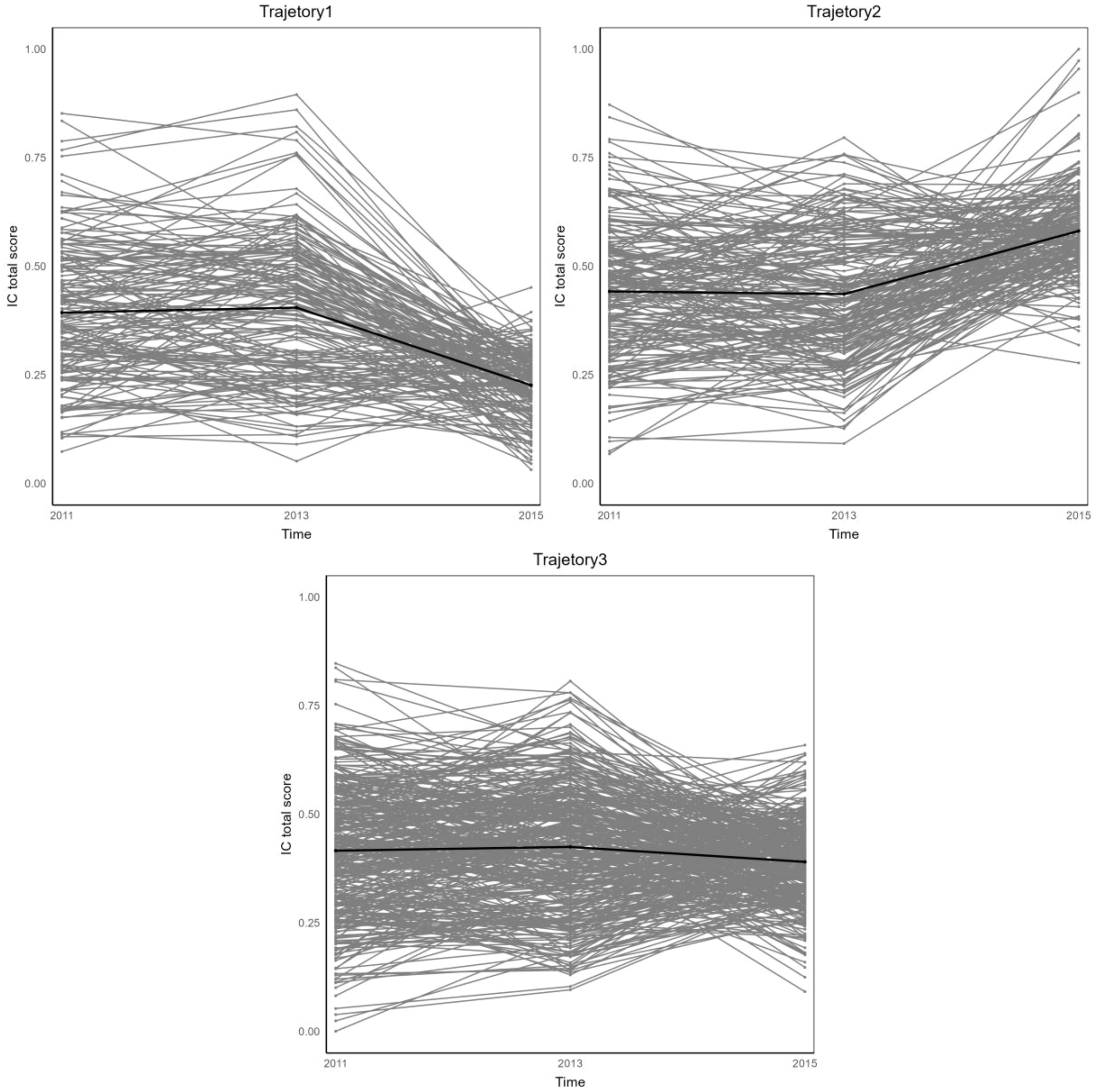
Group estimated mean and estimated individual values for trajectory group.

**Figure 2** illustrates three distinct IC trajectory classes among elderly CVD patients, highlighting significant heterogeneity within the population. **Figure 3** expands on this visualization by incorporating 95% confidence intervals (indicated by color-shaded bands), with spaghetti plots demonstrating acceptable inter-trajectory divergence, supporting the model’s validity. Although individual patients did not follow identical developmental patterns, the trajectories remained relatively stable over the 5-year observation period. As illustrated in **Figure 4**, black lines represent class-specific means, while thin lines depict model-estimated individual trajectories within each class. Class 1 (n = 162, 23.11%) displayed low baseline IC levels with a gradual decline over time, labeled as the Low-decreased Group. Class 2 (n = 196, 27.96%) maintained high baseline IC levels with stable progression, designated as the High-increased Group. Class 3 (n = 343, 48.93%) demonstrated moderate IC levels with minimal longitudinal fluctuations, termed the Medium-stable Group. The alignment between trajectory profiles (class means) and individual trajectories (spaghetti plots) validates the robust goodness-of-fit and the clinical interpretability of the 3-class model.

#### 3.2.2 Sensitivity analysis

In elderly CVD patients, censoring or truncation due to mortality events is inherently correlated with levels of IC depletion. Attrited participants may disproportionately include individuals with accelerated functional decline, potentially introducing estimation bias. Furthermore, the direct exclusion of cases with incomplete observations may be connected with systematic bias. To validate the robustness of trajectory estimations, this study conducted the following supplementary analyses:

##### 3.2.2.1 Attrition bias analysis

**Figure 5:**
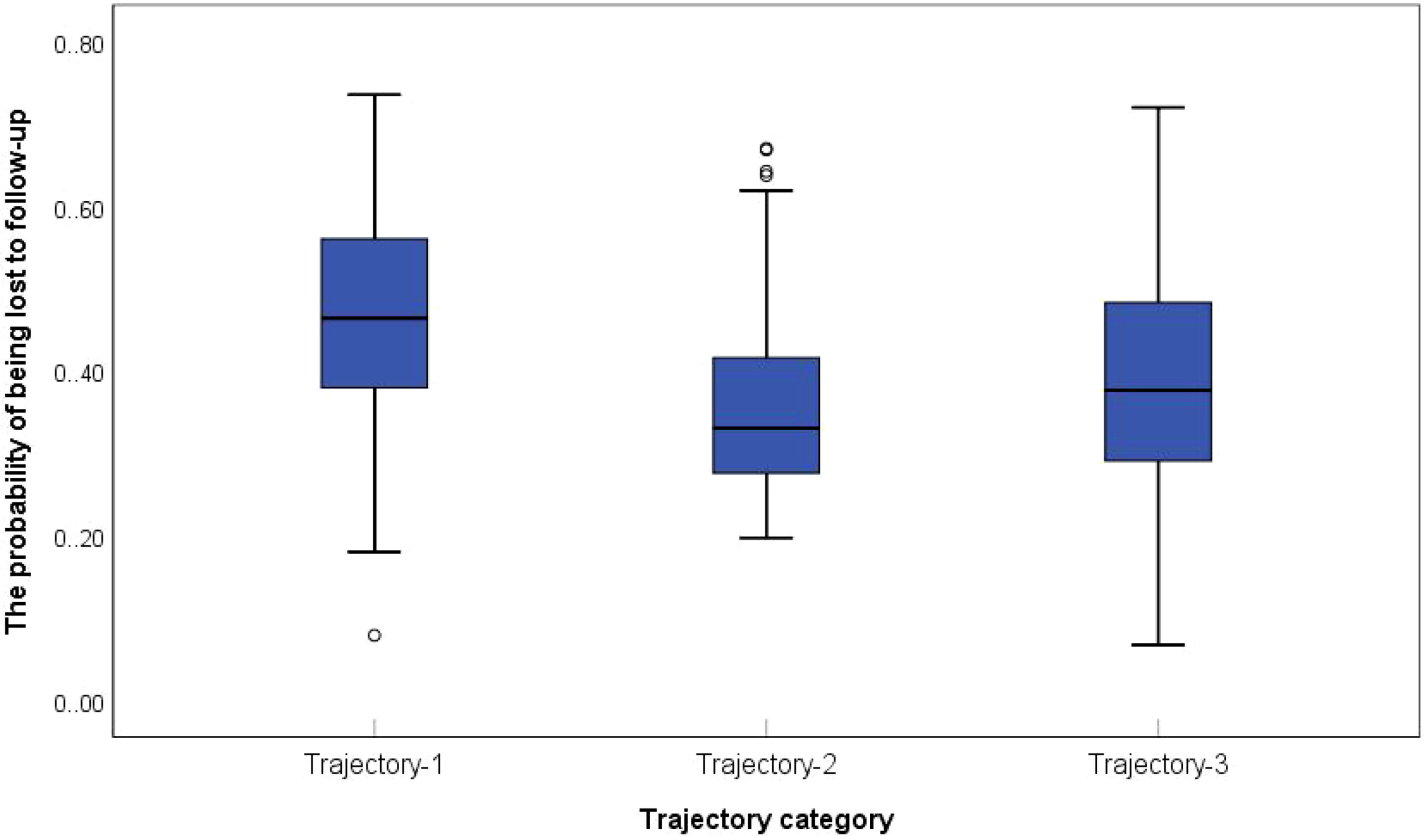
Probability of withdrawal/disappearance of elderly CVD patients with different IC trajectories.

This study employed logistic regression models to predict the probability of withdrawal or loss to follow-up among participants in the 2013 and 2015 final samples. Box plots were generated with trajectory classes 1–3 on the x-axis and attrition probability on the y-axis. The comparative analysis (**Figure 5**) revealed that the Low-decrease Group exhibited the highest attrition rate, followed by the Medium-stable Group. In contrast, the High-increased Group displayed the lowest attrition probability, a pattern that aligns with clinical observations: individuals with severe IC impairment are more likely to withdraw due to health deterioration, mobility limitations, or mortality. These findings confirm that older adults with multi-domain IC deficits are disproportionately susceptible to study withdrawal, which is related to reduced representation of the Low–decreased subgroup in the retained elderly CVD cohort. The accelerated decline observed among attrited Low-decreased cases likely represents extreme scenarios, without fundamentally altering the overall population-level trend directions. Moreover, t he low attrition rate and narrow confidence intervals in the High-increased Group further enhance the reliability of the results. Additionally, GBTM demonstrates robustness against Medium-stable attrition and measurement errors, particularly when trajectory morphologies are distinct and within-class homogeneity is high This ensures that core group integrity is maintained despite attrition (32).

##### 3.2.2.2 Inverse probability weighting

For participants across all three survey waves (2011, 2013, 2015), the inverse probabilities of completing 2013/2015 follow-ups were estimated using logistic regression, with covariates including age, sex, baseline chronic disease status. IPW-adjusted analyses were performed to mitigate non-response and attrition bias. To assess the impact of these adjustments, trajectory class proportions before and after IPW were compared (**Table 5**). The results showed that the absolute changes in class proportions post-adjustment were ≤4%. Despite the higher attrition rates in the Low-decreased Group (**Figure 5**), IPW-adjusted trajectory distributions revealed no statistically significant shifts. This outcome supports the robustness of IC dynamic patterns and their associated factors even under attrition bias.

**Table 5:** Comparison of trajectory class proportions pre- and post-Adjustment.

Trajectory Group	Original Model	IPW-Adjusted Model	△%
Trajectory-1	23.11%	26.89%	+3.78%
Trajectory-2	48.93%	47.97%	-0.96%
Trajectory-3	27.96%	25.13%	-2.83

##### 3.2.2.3 Distribution-based quantification of effect size differences across trajectory groups

To validate the trajectory classification’s validity and clinical significance, this study implemented distribution-based effect size analyses to assess model sensitivity. Following Cohen’s d thresholds (33), where *d* ≥ 0.2 indicates small effects, *d* ≥ 0.5 moderate effects, and *d* ≥ 0.8 large effects, pairwise effect sizes were calculated between the three trajectory groups, as shown in **Table 6**. All inter-group differences reached the large-effect threshold (*d* ≥ 0.8), indicating substantial health status disparities that confirm the rationale behind the classification. Specifically, the comparison between the Low-decreased Group and Medium-stable Group yielded a large effect (*d* = 0.831), reinforcing a clinically meaningful divergence. Similarly, the difference between the Medium-stable Group and High-increased Group difference exceeded the large-effect threshold (*d* = 0.936), highlighting the need for early interventions for the medium-stable subgroup to mitigate functional deterioration.

**Table 6:** Effect size gradients and clinical implications of IC trajectory differences.

Trajectory Group Comparison	Cohen’s *d*	Effect Size Magnitude
Trajectory-1 *vs* Trajectory-2	1.052	Large Effect
Trajectory-1 *vs* Trajectory-3	0.831	Large Effect
Trajectory-2 *vs* Trajectory-3	0.936	Large Effect

### 3.3 Analysis of associated factors of the IC trajectory in elderly patients with CVD

#### 3.3.1 Association of time-invariant variables (static characteristics) with trajectory classification

Considering the potential interplay between socioeconomic/environmental factors and intrinsic capacity (IC) trajectories in elderly cardiovascular patients, this section systematically explores the statistical associations between time-invariant baseline characteristics and trajectory group membership. The time-invariant features included: (1) sociopolitical environment and residential conditions; (2) cumulative lifestyle habits throughout adulthood (such as smoking, alcohol consumption, napping, and dietary patterns); and (3) demographic factors (age, sex, geographic residency, and educational attainment). Univariate analyses revealed significant intergroup heterogeneity in IC trajectory classification across the following indicators: educational level (*χ* ^2^ = 15.005, *P=*0.020), geographic residency (*χ* ^2^ = 34.343, *P*<0.001), housing type (*χ* ^2^ = 17.548, *P*<0.001), sanitation facilities (*χ* ^2^ = 33.471, *P*=0.001), cooking fuel type (*χ* ^2^ = 25.595, *P*<0.001), and heating systems (*χ* ^2^ = 21.595, *P*<0.001). Detailed univariate results are provided in **Supplementary Table S1**.

Variables that demonstrated statistical significance in univariate analyses were included as independent variables in multinomial logistic regression, with IC trajectory classification as the dependent variable (reference group: Class 2 - High-increased Group). The results (**Table 7**) revealed the following: Compared to Class 2 (High-increased Group), Class 1 (Low-decreased Group) showed higher odds of rural residence (*OR* = 1.796, *P* = 0.040), non-flush toilet facilities (*OR* = 1.890, *P* = 0.039), and solid fuel dependence (*OR* = 0.632, *P* = 0.086). Class 3 (Medium-stable Group) exhibited a higher likelihood of primary/no formal education (*OR* = 2.442, *P* = 0.094), rural residency (*OR* = 1.718, *P* = 0.021), and non-flush toilet access (*OR* = 1.846, *P* = 0.012).

**Table 7:** Association results of time-invariant variables on IC trajectory grouping in elderly CVD patients.

Variables	Trajectory 1		Trajectory 3	
	*OR*	*Se*	*OR*	*Se*
Educational status (ref. = College or university)				
No formal education or primary	0.915	0.584	2.442 *	0.533
Junior high school	0.723	0.573	2.061	0.521
Senior high school	0.771	0.561	1.821	0.512
Residence region (ref. = Cities and towns)				
Villagers	1.796 **	0.285	1.718 **	0.234
Housing type (ref. = Multi-story building)				
One-story building	1.201	0.275	0.712	0.227
Toilet type (ref. = Toilet with a seat)				
Toilet without a seat	1.890 **	0.309	1.846 **	0.244
Main source of cooking fuels (ref. = Solid fuels)				
Clean fuels	0.632 *	0.268	0.859	0.228
Heating equipment (ref. = No)				
Yes	0.887	0.352	0.811	0.278

*, **, *** indicate significance at the 10%,5%, and 1% levels, respectively.

#### 3.3.2 Association of time-varying variables with trajectory morphology

As previously mentioned, time-invariant characteristics (e.g., endowments, resource allocation) during the geriatric transition may exhibit statistical associations with IC trajectory classification. In contrast, time-varying variables might dynamically correlate with specific trajectory morphologies. To this end, we investigated dynamic associations between time-varying covariates and the trajectory-specific developmental patterns across different subgroups. The time-varying covariates included health status indicators, social engagement diversity and marital status. Using three-wave panel data, we employed panel regression models to analyze the dynamic relationships between these variables and IC scores (i.e., trajectory-specific developmental patterns). The results are detailed in **Table 8**.

**Table 8:** Association results of time-varying variables on trajectory patterns across IC subgroups in elderly CVD patients.

Variables	Trajectory 1		Trajectory 2		Trajectory 3	
	*Coe.*	*Se.*	*Coe.*	*Se.*	*Coe.*	*Se.*
Marital status	-0.035	0.023	0.045 **	0.019	0.023	0.014
Major accidental injury	0.007	0.026	0.024	0.024	0.005	0.016
Toothless	-0.044 **	0.020	-0.005	0.019	-0.033 **	0.015
Chronic pain	-0.022 *	0.011	-0.003	0.011	-0.032 ***	0.008
Sleep disorders	-0.023	0.014	0.002	0.015	-0.019 *	0.011
Richness of social activities	0.015 ***	0.005	0.012 **	0.004	0.018 ***	0.004
Chronic disease co-morbidity level	-0.004	0.006	-0.009 *	0.005	-0.007 **	0.003
_cons	0.437 ***	0.028	0.409 ***	0.024	0.415 ***	0.017

*, **, *** indicate significance at the 10%,5%, and 1% levels, respectively.

As illustrated in **Table 8**, marital status displayed significant heterogeneity in its relationship with IC scores among elderly CVD patients. A strong positive correlation was observed in the High-increased Group(*Coe*=0.045, *P*=0.016), while a non-significant positive trend was noted in the Medium-stable Group (*Coe*=0.023, *P*=0.116). In contrast, a non-significant negative trend was found in the Low-decreased Group (*Coe*=-0.035, *P*=0.121). Major accidental injuries did not show any statistically significant associations with IC scores across all trajectory groups (*Coe_t1_ =* 0.007, *P*=0.762; *Coe_t2_ =* 0.024, *P*=0.316; *Coe_t3_ =* 0.005, *P*=0.756). Dental loss demonstrated significant negative associations in both the Medium-stable Group (*Coe*=-0.033, *P*=0.027) and Low-decreased Group (*Coe*=-0.044, *P*=0.028), with stronger effects observed in the latter. Chronic pain was negatively associated with IC scores, achieving statistical significance in the Medium-stable Group (*Coe*=-0.032, *P*<0.001) and marginal significance in the Low-decreased Group (*Coe*=-0.022 *P*=0.063), though no significant association was found in the High-increased Group (*Coe*=-0.003, *P*=0.774). Sleep disturbances approached statistical significance only in the Medium-stable Group (*Coe*=-0.019, *P*=0.083). Multimorbidity severity had a negative impact on IC scores in the High-increased Group (*Coe*=-0.009, *P*=0.079) and a significant negative effect in the Medium-stable Group (*Coe*=-0.007, *P*=0.042). Active social participation strongly correlated with IC maintenance/improvement, showing robust effects in the Medium-stable Group (*Coe*=0.018, *P*<0.001) and Low-decreased Group (*Coe*=0.015, *P*=0.008).

## 4 Discussion

### 4.1 Elderly patients with CVD demonstrate different trajectories of IC

This study identified three latent trajectory classes of intrinsic capacity (IC) among elderly cardiovascular disease patients ultilizing GBTM: Low-decreased Group (23.11%), High-increased Group (27.96%), and Medium-stable Group (48.93%). The results indicate that, while the overall IC development in elderly CVD patients tends to be stable (represented primarily by the Medium-stable Group), substantial population heterogeneity is observed across trajectory patterns. It is noteworthy that the classification and trends of IC trajectories in this CVD cohort differ markedly from existing findings in general elderly populations. Previous studies have predominantly reported universal IC decline trajectories in older adults (14, 34, 35). In contrast, our results reveal a significant proportion (48.93%) maintaining stability, which can potentially be attributed to the study’s focus on CVD patients with systemic impairments. Baseline data indicate that this population exhibits multi-domain IC deficits upon enrollment, resulting in lower baseline IC levels compared to community-dwelling older adults. Consequently, the limited residual decline capacity may be responsible for the observed stabilization in longitudinal trajectories.

The Low-decreased Group was characterized by sustained functional deterioration, attributable to complex pathophysiological underpinnings, uncontrolled comorbidities, and insufficient therapeutic interventions. This subgroup represents a high-risk population that should be prioritized for rehabilitation. Previous investigations have highlighted the coexistence of progressive deterioration and reversible trajectories in cardiovascular disease progression (36, 37), emphasizing the necessity of identifying modifiable factors for clinical intervention.

The High-increased Group, distinguished by superior baseline function and improvement trends that counter disease progression, demonstrates that IC deficits can be reversed through intensified treatment and sustained health management. These findings indirectly validate the efficacy and necessity of tertiary prevention strategies in cardiovascular care.

As the largest subgroup (48.93%), the Medium-stable Group maintained relatively consistent IC levels throughout the follow-up period, suggesting that the majority of elderly CVD patients have transitioned into a chronic adaptation phase wherein physiological self-regulation buffers against disease progression. This trajectory indicates optimal cost-effectiveness for targeted interventions aimed at reducing disease burden. While current medical supports (e.g., pharmacotherapy, basic care) demonstrate functional preservation effects, clinicians must remain vigilant regarding potential decompensation risks that may be masked by apparent stability, particularly when patients are exposed to external stressors.

Although limited by CHARLS data availability which constrained follow-up duration, this study reveals critical heterogeneity in IC trajectories among elderly CVD patients and provides evidence-based foundations for mid-term (4–6 years) health management. Future research should aim to validate long-term trajectory patterns through extended observation periods.

### 4.2 Association of time-invariant characteristics with IC trajectories in elderly CVD patients

This investigation revealed systematic associations between IC trajectory classification and socioeconomic-environmental fators among elderly cardiovascular patients. Rural residence demonstrated elevated odds ratios (OR) for both the Low-decreased Group and Medium-stable Group, potentially related to urban-rural disparities in healthcare resource allocation and limited accessibility to health services. The comparative scarcity of specialized medical support and chronic disease management systems in rural areas may limit the effectiveness of early disease intervention. Delayed emergency response times further exacerbate challenges in functional recovery (38, 39). The “empty-nest” phenomenon, predominantly observed in rural regions, weakens social support networks, thereby depriving patients of essential care resources and emotional sustenance during disease adaptation. Such social disconnection may impair functional compensatory capacity through psycho-neuroendocrine mechanisms (40).

Elderly CVD patients residing in single-story dwellings demonstrated comparatively higher IC levels than those in multi-story apartments. This disparity may be associated with specific environmental advantages: ground-floor living eliminates mobility barriers, thereby facilitating engagement in daily functional activities (41), while increased exposure to natural light and green spaces has been shown to modulate somatic function through neuroendocrine pathways, as evidenced by previous research (42). These findings underscore the necessity for clinicians to systematically evaluate residential environments. Tailored home-based exercise regimens (e.g., treadmill walking, stretching) and caregiver-facilitated outdoor activities could expand patients’ utilization of living space, consequently enhancing functional engagement.

The absence of flush toilets demonstrated elevated OR in both the Low-decreased Group and Medium-stable Group. Inadequate sanitation facilities may exacerbate functional barriers in daily activities—for instance, squatting-induced postural changes increase cardiac preload, thereby intensifying cardiovascular burden. Age-related declines in muscle strength and balance control render older adults vulnerable to instability during squatting, as maintaining a low center of gravity necessitates sustained postural stability, significantly elevating fall risks. Studies indicate that toilet-related falls constitute a prevalent cause of geriatric injuries in China, with squat toilets directly compromising mobility through fall-induced trauma (43, 44). These findings suggest that suboptimal sanitation environments not only accelerate functional decline but also constrain compensatory plasticity by limiting functional recovery potential. Consequently, environmental modifications within comprehensive disease management must concurrently address dual objectives: risk mitigation (e.g., fall prevention) and functional enhancement (e.g., adaptive equipment provision).

The association between solid fuel use and trajectory grouping may elucidate complex equilibrium mechanisms operating between environmental exposure and individual adaptability. In the High-increased Group, the maintenance of functional homeostasis among solid fuel users might relate to their comprehensive compensatory capacity: (1) behavioral adaptations (e.g., improved ventilation, clean energy adoption) may reduce actual exposure risks, while enhanced health literacy facilitates early symptom recognition and healthcare utilization, thereby partially offsetting environmental hazards; (2) inherent biological resilience may delay cumulative pollutant-induced damage to cardiovascular metabolic pathways (45). However, when exposure duration/dose exceeds physiological regulatory thresholds, protective buffering effects are diminished, resulting in functional stabilization rather than continued improvement.

Furthermore, this study identified a significant positive correlation between educational attainment and IC levels, with lower education groups being disproportionately represented in the Low-decreased trajectory. These findings are consistent with Magnani et al.’s research demonstrating a dose-response relationship between education and lifetime CVD risk (46), where higher education correlates with improved prognosis through health behavior mediation effects (46). As a fundamental social factor of health, education is linked to functional maintenance in CVD patients via multiple pathways, including health literacy enhancement and lifestyle optimization. Chinese cohort data reveal elevated smoking rates and risky alcohol consumption in populations with lower educational attainment, providing mechanistic insights into the education-behavior-IC nexus (47). This phenomenon reflects the multilevel effect of social healthfactors: education, as a structural socioeconomic status indicator, not only correlates with health resource accessibility but also cumulatively shapes health information processing capacity (48). Consequently, CVD prevention systems should prioritize addressing healthcare access barriers in populations with limited educational attainment through: Targeted health literacy programs integrated within primary care services; Differentiated medical subsidy policies; and optimized community health communication. These measures aim to mitigate health inequities rooted in educational disparities.

### 4.3 Association of time-varying characteristics with IC trajectory morphology in elderly CVD patients

This investigation elucidated trajectory-dependent associations between marital status and IC scores: a significant positive correlation was observed in the High-increased Group, a weak positive association in the Medium-stable Group, and a non-significant negative tendency in the Low-decreased Group. These findings underscore the health-promoting effects of marital support. High-functioning patients may benefit from spousal collaboration in health management (e.g., medication adherence reminders, exercise companionship). In contrast, in the Low-decreased Group, caregiving burdens inherent to marital relationships may partially offset the supportive benefits. This phenomenon aligns with Antonucci’ s social Convoy model, which suggests that the protective effects of social ties dynamically modulate according to individuals’ baseline health status (49, 50). These results emphasize the importance of assessing marital quality in medium- and low-functioning subgroups, providing respite care services for solitary or care-overburdened households, and developing couple-based health management programs to optimize the efficacy of marital support.

The significant subgroup heterogeneity in the association between dental loss and IC. It emerged as an independent risk factor in both the Medium-stable Group (moderate functional reserve) and Low-decreased Group (compromised baseline function), with a 38% increase in effect size in the latter. Reduced masticatory efficiency may impair protein intake, leading to an anabolic-catabolic imbalance in muscles, thereby exacerbating declines in vitality and mobility. Substantial evidence links oral health to cardiovascular outcomes, with each lost tooth increasing coronary heart disease risk by 1.04% (51). Notably, tooth loss is connected with cardiovascular morbidity in older U.S. adults (52), and dental attrition may serve as an auxiliary predictor of cardiovascular risk (53). These findings advocate for the inclusion of masticatory function assessment into routine cardiovascular rehabilitation protocols, with a focus on masticatory training and probiotic interventions in medium- and low-functioning subgroups to mitigate degenerative cascades.

Sleep disturbances demonstrated a marginally significant negative association with IC in the Medium-stable Group, potentially indicating compensatory adaptations in sleep regulation. This subgroup, existing at a functional compensation threshold, may exhibit behavioral trade-offs in sleep architecture. Disruptions in circadian rhythm due to sleep disorders likely impair cardiovascular stress adaptation through pathways involving inflammatory cytokines and autonomic nervous system balance indices (54). Furthermore, sleep deprivation or chronodisruption may dysregulate ghrelin-leptin signaling, exacerbating lipid metabolism disturbances and elevating risks of dyslipidemia, obesity, and related cardiovascular sequelae (55). Clinicians should prioritize sleep hygiene interventions in the Medium-stable Group to stabilize biological rhythms and prevent functional decline tipping points.

This investigation identified a significant negative correlation between the number of chronic diseases and IC levels in elderly CVD patients. These findings are consistent with those of Ding Hua et al. (56), who identified the coexistence of multiple diseases as a risk factor for the developmental trajectory of debilitation. Elderly patients with CVD experience prolonged chronic inflammation, predisposing them to the coexistence of multiple morbidities. The presence of these additional chronic conditions contributes to functional impairment across various tissues and organs, potentially accelerating the decline of physiological functions in these patients. This decline diminishes the body’s stress resilience, ultimately compromising IC. Furthermore, the coexistence of multiple diseases necessitates the administration of various medications for their management. This polypharmacy might be related to adverse drug effects and interactions, potentially causing harm to metabolic organs such as the liver and kidneys. Additionally, it may diminish the body’s resilience to respond to external stimuli, accelerating the decline of IC (57). This highlights the need for healthcare professionals to prioritize the consideration of multimorbidity and multidrug interactions in the management of elderly patients with CVD experiencing multiple chronic disease comorbidities.

This study demonstrated a significant association between chronic pain and declining IC trajectories, which is consistent with IC’s established role in reflecting the systemic reserve capacity of older adults (15). CVDs exhibit pathological associations with various pain syndromes, including peripheral arterial disease, angina pectoris, thoracic outlet syndrome, post-amputation pain, complex regional pain syndrome, and post-stroke pain (58). Prior research has also demonstrated that older adults experiencing chronic pain are at an elevated risk of debilitation (59). Pain and frailty may share common underlying physiological mechanisms, such as inflammatory markers. The concept of ‘inflammatory aging’ describes the altered immune function observed in elderly patients with CVD, which is associated with reduced IC (60). Furthermore, the pain imbalance theory posits that chronic pain acts as a stressor, hastening the IC decline among the elderly (61). Alterations in the emotional pathways common to both chronic pain and depression may correlates with analogous abnormalities in information processing. Patients enduring chronic pain over extended durations are susceptible to developing anxiety and depression, which subsequently contribute to impairments in IC. Consequently, in the formulation of a comprehensive intervention and rehabilitation program for elderly patients with CVD, it is imperative to incorporate pain management strategies alongside psychological interventions. This integrated approach aims to holistically improve the patients’ IC.

This investigation demonstrated a significant association between diversified social participation and decelerated decline in IC among elderly CVD patients, which aligns with the social engagement-driven health preservation paradigm proposed by Wang Haiyan’s research team (62). WHO (4) posits that health in older age represents a dynamic process rather than a static state. Even minor alterations in functional capacity and environmental factors can have significant long-term implications, emphasizing the importance of interactions between individuals and their environments. Social participation serves as a primary avenue for older adults to engage in social interactions, wherein they are afforded a variety of social roles that leverage their age-related strengths. This engagement contributes to the enhancement of their self-esteem and self-confidence while also facilitating improvements in cognitive function and psychological well-being to some extent (63). Older adults strategically direct their attention and energy toward interpersonal interactions and social activities as a means to mitigate cognitive decline and, to some degree, diminish the adverse effects of cardiovascular events. Research indicates that decreased cardiovascular stress reactivity correlates with lower levels of social participation (64). Healthcare professionals are encouraged to prioritize the development and implementation of management strategies addressing social isolation among elderly patients with CVD. These strategies may include peer support initiatives, team-based psychological interventions, social network enhancement programs, and animal-assisted therapies.

## 5 Conclusions and limitations

This study represents a longitudinal investigation into the IC trajectories of elderly CVD patients in China. Three distinct IC trajectory classes were identified, revealing inter-individual variability in IC progression, although the majority of patients maintained relatively stable IC status throughout the follow-up period. The trajectory classification was related to baseline characteristics and time-varying covariates. Factors such as education level, geographic residency, housing type, sanitation facilities, and household energy sources demonstrated significant trajectory-specific associations, likely shaped by disparities in healthcare accessibility and cumulative environmental exposures. Time-varying factors, including marital status, masticatory function, sleep disturbances, chronic pain, multimorbidity, and social engagement diversity, were associated with trajectory changes in IC evolution.

However, several limitations should be noted: (a) Self-reported data may introduce recall bias, potentially compromising the accuracy of IC assessments. Future studies should incorporate objective biomarkers to enhance validity. (b) Selective loss to follow-up, particularly among severely impaired individuals, may underestimate the proportion of low-functioning patients. (c) The 5-year follow-up of 701 participants from public databases may not fully capture long-term intra-individual IC fluctuations, particularly in relation to late-life health deterioration dynamics. Extended cohorts with broader geographic and temporal coverage are necessary. (d) As a retrospective study using group-based trajectory modeling (GBTM), a descriptive tool, the identified correlates, but it cannot establish causality. Causal inference methods are required for mechanistic validation in the future research. (e) The findings are contextually bound to China’s healthcare system, sociocultural norms, and support networks. Multinational studies are essential to differentiate universal IC trajectory factors from region-specific drivers, which would inform the development of stratified global aging interventions.

## Data Availability

The original contributions presented in the study are included in the article/**Supplementary Material**. Further inquiries can be directed to the corresponding author.
