# Supplementary material for: The trajectory of intrinsic capacity and its related factors among elderly Chinese patients with cardiovascular disease: a prospective cohort study
Source: Front Endocrinol (Lausanne). 2025 May 27;16:1539982. doi: 10.3389/fendo.2025.1539982 (PMC12148865; doi:10.3389/fendo.2025.1539982)
Supplement: Supplementary file 1 [file Table1.docx]

**Table S1** Univariate analysis of IC trajectories in elderly patients with CVD

|  | Characteristic | All participants (n=701) | Trajectory 1  (n=162) | Trajectory 2  (n=196) | Trajectory 3  (n=343) | *X^2^/F* | *P-value* |
| --- | --- | --- | --- | --- | --- | --- | --- |
| Essential information | **Age** (x±s) | 66.19±5.134 | 65.46±4.282 | 66.51±5.683 | 66.36±5.155 | 2.214 | 0.110 |
|  | **Gender** |  |  |  |  | 0.318 | 0.853 |
|  | Male | 402 | 96 | 111 | 195 |  |  |
|  | Female | 299 | 66 | 85 | 148 |  |  |
|  | **Educational status** |  |  |  |  |  |  |
|  | No formal education or primary | 215 | 56 | 45 | 114 | 15.005 | 0.020 |
|  | Junior high school | 237 | 52 | 64 | 121 |  |  |
|  | Senior high school | 224 | 48 | 75 | 101 |  |  |
|  | College or university | 25 | 6 | 12 | 7 |  |  |
|  | **Residence region** |  |  |  |  | 34.343 | < 0.001 |
|  | Villagers | 436 | 118 | 89 | 229 |  |  |
|  | Cities and towns | 265 | 44 | 107 | 114 |  |  |
|  | **Marital status** |  |  |  |  | 0.738 | 0.691 |
|  | Married | 596 | 139 | 163 | 294 |  |  |
|  | Not married | 105 | 23 | 33 | 49 |  |  |
| Healthy Lifestyle Habits | **Napping habit** |  |  |  |  | 0.536 | 0.765 |
|  | Yes | 470 | 112 | 132 | 226 |  |  |
|  | No | 231 | 50 | 64 | 117 |  |  |
|  | **Dietary habits** |  |  |  |  | 0.407 | 0.816 |
|  | 3 meals per day | 640 | 146 | 179 | 315 |  |  |
|  | Others | 61 | 16 | 17 | 28 |  |  |
|  | **Smoking history** |  |  |  |  | 2.287 | 0.319 |
|  | Yes | 315 | 72 | 80 | 163 |  |  |
|  | No | 386 | 90 | 116 | 180 |  |  |
|  | **Drinking** |  |  |  |  | 6.213 | 0.184 |
|  | None of these | 474 | 117 | 131 | 226 |  |  |
|  | Drink more than once a month | 182 | 36 | 47 | 99 |  |  |
|  | Drink but less than once a month | 45 | 9 | 18 | 18 |  |  |
|  | **Richness of social activities** |  |  |  |  | 8.777 | 0.187 |
|  | 0 | 251 | 61 | 74 | 116 |  |  |
|  | 1 | 281 | 70 | 67 | 144 |  |  |
|  | 2 | 128 | 23 | 38 | 67 |  |  |
|  | 3 or more | 41 | 8 | 17 | 16 |  |  |
| Social security | **Health insurance** |  |  |  |  | 0.258 | 0.879 |
|  | Yes | 675 | 157 | 188 | 330 |  |  |
|  | No | 26 | 5 | 8 | 13 |  |  |
|  | **Pension insurance** |  |  |  |  | 1.150 | 0.563 |
|  | Yes | 153 | 38 | 46 | 69 |  |  |
|  | No | 548 | 124 | 150 | 274 |  |  |
| Residential environment | **Housing type** |  |  |  |  | 17.548 | < 0.001 |
|  | One-story building | 405 | 114 | 95 | 196 |  |  |
|  | Multi-story building | 296 | 48 | 101 | 147 |  |  |
|  | **Toilet type** |  |  |  |  | 33.471 | < 0.001 |
|  | Toilet without a seat | 514 | 134 | 114 | 266 |  |  |
|  | Toilet with a seat | 187 | 28 | 82 | 77 |  |  |
|  | **Main source of cooking fuels** |  |  |  |  | 25.595 | < 0.001 |
|  | Clean fuels | 365 | 64 | 129 | 172 |  |  |
|  | Solid fuels | 336 | 98 | 67 | 171 |  |  |
|  | **Heating equipment** |  |  |  |  | 21.095 | < 0.001 |
|  | Yes | 126 | 20 | 56 | 50 |  |  |
|  | No | 575 | 142 | 140 | 293 |  |  |
|  | **Interior temperature** |  |  |  |  | 4.744 | 0.315 |
|  | Hot | 57 | 13 | 11 | 33 |  |  |
|  | Bearable | 630 | 114 | 183 | 303 |  |  |
|  | Cold | 14 | 5 | 2 | 7 |  |  |
|  | **Handicapped facility** |  |  |  |  | 0.954 | 0.621 |
|  | Yes | 188 | 47 | 48 | 93 |  |  |
|  | No | 513 | 115 | 148 | 250 |  |  |
| Health status | **Chronic disease co-morbidity level** |  |  |  |  | 9.412 | 0.152 |
|  | 1 | 171 | 34 | 49 | 88 |  |  |
|  | 2 | 232 | 53 | 53 | 126 |  |  |
|  | 3 | 143 | 39 | 46 | 58 |  |  |
|  | 4 or more | 155 | 36 | 48 | 71 |  |  |
|  | **Major accidental injury** |  |  |  |  | 4.571 | 0.102 |
|  | Yes | 65 | 11 | 14 | 40 |  |  |
|  | No | 636 | 151 | 182 | 303 |  |  |
|  | **Chronic pain** |  |  |  |  |  |  |
|  | Yes | 480 | 62 | 53 | 106 | 5.303 | 0.071 |
|  | No | 221 | 100 | 143 | 237 |  |  |
|  | **Toothless** |  |  |  |  |  |  |
|  | Yes | 75 | 18 | 26 | 31 | 2.370 | 0.306 |
|  | No | 626 | 144 | 170 | 312 |  |  |
|  | **Sleep disorders** |  |  |  |  |  |  |
|  | Yes | 136 | 41 | 38 | 57 | 5.315 | 0.070 |
|  | No | 565 | 121 | 158 | 286 |  |  |
